# Students’ perceptions of learning environment and their leisure-time exercise in medical school: Does sport background matter?

**DOI:** 10.1007/s40037-020-00560-w

**Published:** 2020-02-03

**Authors:** Oksana Babenko, Amber Mosewich, Janelle Sloychuk

**Affiliations:** 1 Faculty of Medicine & Dentistry, University of Alberta, Edmonton, Alberta Canada; 2 Faculty of Kinesiology, Sport, and Recreation, University of Alberta, Edmonton, Alberta Canada

**Keywords:** Medical students, Sport background, Basic psychological needs, Leisure-time exercise

## Abstract

**Introduction:**

The effectiveness of medical school efforts in addressing suboptimal student wellbeing rests, in part, on how students perceive their learning environment. The study aim was to determine whether students’ sport background was a contributing factor in students’ perceptions of the medical program as supportive of their basic psychological needs for autonomy, competence, and relatedness. We also examined the relationship between sport background and students’ leisure-time exercise in medical school.

**Methods:**

Using an online questionnaire, quantitative data were collected from students enrolled in the 4‑year medical program at a large Canadian university. Two hundred (*n* = 200) students had complete responses on the measures used in the study. Analysis of variance and correlational analysis were used to examine the relationships between students’ sport background, their perceptions of the learning environment, and leisure-time exercise in medical school.

**Results:**

Compared with students with no sport background, students with a team sport background perceived their need for relatedness to be satisfied to a greater degree in the medical program. Students who pursued sports at higher levels of involvement (competitively) perceived the medical program as more autonomy-supportive than students who pursued sports at lower levels of involvement (recreationally). Irrespective of their sport background, students’ involvement in leisure-time exercise decreased over the years in the medical program. However, students with a sport background engaged in leisure-time exercise in medical school to a greater extent than students with no sport background.

**Discussion:**

The findings indicate that sport background is associated with students’ perceptions of the learning environment as supportive of their needs for autonomy and relatedness, but not for competence, and is linked to their leisure-time exercise in medical school. The observed relationships could help inform medical schools curricular initiatives in preventing student burnout right from the start of medical school.

## Introduction

Medical schools have been tasked with addressing concerns around student wellbeing [1, 2]. Compared with their peers in the general population, medical students experience substantially higher levels of psychological distress throughout the course of their studies [3, 4]. The effectiveness of medical school efforts to create optimal learning environments depends, in part, on how students perceive the environment around them [5].

According to Ryan and Deci’s self-determination theory [6, 7], when people perceive that their basic psychological needs for autonomy, competence and relatedness are supported in the environment, they experience personal and professional wellbeing [6–10]. The need for autonomy is defined as people’s desire to have control over their lives, have a choice and make decisions [6, 7]. The need for competence is the desire to acquire new knowledge and skills and to feel capable of solving problems [6, 7]. The need for relatedness is the desire to belong and feel connected with others [6, 7]. Fulfilling these fundamental psychological needs, however, can be a challenge in medical school. Medical training is abundant with rules and dos and don’ts for learners, with little leeway. Various hierarchies and administrative regulations pose challenges to medical learner inclusiveness in the professional community, resulting in feelings of isolation. The need for competence is challenged during a long period of medical training in which learners are continuously labeled as insufficiently competent for unsupervised practice [11].

In spite of the potentially psychologically challenging environment, it appears that some students, specifically students with a sport background, are able to respond adaptively, resulting in positive learning and wellbeing outcomes [12, 13]. For example, Strowd and colleagues [12] found that students who participated in collegiate varsity athletics excelled in medical school, outperforming their peers in both standardized tests and clinical clerkship environment. Our research with medical students revealed that students who had participated in high-stakes athletic training and competition during their formative years had better academic wellbeing than their peers who had no such experience [13]. Specifically, applying achievement goal theory [14], we observed that students with a competitive sport background had lower academic burnout scores and endorsed avoidance goals (maladaptive forms of motivation) to a lesser degree than students without such a background [13]. A possible explanation for the observed outcomes is that involvement in sport provides opportunities for young people to develop abilities to perform under pressure, respond positively to criticism and feedback, and embrace failures as opportunities for further development [15], attributes that they subsequently can draw upon in college or university [12]. In light of the findings that students with a sport background tend to experience better academic wellbeing and excel in medical school [12, 13], the question arises as to how these students possibly perceive and approach the learning environment they are in that may enable them to respond to challenges of medical training adaptively.

As such, guided by self-determination theory [6, 7], the primary objective of this study was to investigate the relationship between students’ sport background and their perceptions of the learning environment. Specifically, we investigated whether the level and the type of sport in which students had participated prior to medical school were contributing factors in students’ perceptions of the medical program as supportive of their needs for autonomy, competence and relatedness. In addition, we examined the relationship between students’ sport background and their leisure-time exercise in medical school, a well-established factor in student wellbeing [13, 16–18]. Understanding these relationships could help inform curricular initiatives of medical schools in addressing concerns around student wellbeing.

## Methods

This study is part of a larger research program on personal and contextual factors, including sport background, in learning and wellbeing of medical trainees [13, 18–20]. Ethics approval (#Pro00066510) was granted by the Research Ethics Board (REB 2) at the University of Alberta, Canada, prior to data collection. The study was carried out in accordance with the Declaration of Helsinki [21], with no harm to participants, the guaranteed anonymity of participants, and the obtained informed consent of participants.

### Participants

Quantitative data were collected from medical students, using an online questionnaire. Of 267 students who had agreed to participate in the study, 200 students (75%) had complete responses on the measures used in the study: 23% (*n* = 46) of the respondents were in year 1, 30% (*n* = 60) in year 2, 21% (*n* = 42) in year 3, and 26% (*n* = 52) in year 4. Nine students had missing information on their age and/or sex. Of those students who provided their demographic information, 60% (*n* = 116) were female and 95% (*n* = 185) were 20–29 years of age.

### Measures

#### Sport background

Medical students were asked ‘Please indicate the type of sport in which you have participated the longest period of time in your life’, with the following three response options provided: none; team sport; and individual sport. We chose not to provide examples of specific sports and let the students decide if they viewed their sport as an individual or a team sport. Subsequently, students were asked ‘If you selected team or individual sport, please indicate the level of involvement in the sport’, with the following response options provided: 1—recreational non-competitive; 2—competed in intramurals or in a recreational league; 3—competed against athletes from my city/town and nearby communities; 4—competed against athletes from around my province/state/territory; 5—competed against athletes from nearby provinces/states/territories; 6—competed at a National Championship; and 7—competed against athletes from a country other than my own or as a member of a national team. In both questions, students were allowed to choose only one response option.

#### Leisure-time exercise

The Godin Leisure-Time Exercise Instrument [22] was used to measure students’ leisure-time exercise in medical school. Using one of the response options (never; 1–3 times a week; 4–6 times a week; 7 times a week or more), students were asked to indicate the number of times they engaged in mild, moderate, and strenuous leisure-time exercise bouts of at least 15 min duration in a typical week. Examples of such activities were provided for each intensity category. The number of bouts at each intensity was then multiplied by 3, 5, and 9 metabolic equivalents (for mild, moderate, and strenuous activity, respectively) and summed to derive a leisure-time exercise score for each student. Higher scores were indicative of greater involvement in leisure-time exercise.

#### Basic psychological needs

The 12-item Basic Psychological Needs Scale [23] was used to assess levels of satisfaction of each need (autonomy, competence, relatedness; 4 items each) in the learning environment, as perceived by students. Using a Likert-type scale (1—strongly disagree to 6—strongly agree), students were asked to indicate how they typically felt in relation to their medical program. Sample items and internal consistency reliabilities (Cronbach α) of the needs measures in this study were as follows: ‘In my program, I can take on responsibilities’ (autonomy; α = 0.75); ‘In my program, I feel competent’ (competence; α = 0.79); and ‘When I am with the people from my program, I feel I am a friend to them’ (relatedness; α = 0.89). The total scores on each need measure could range from 4 to 24, with higher scores indicating greater satisfaction of the respective needs in the learning environment, as perceived by students.

### Analyses

Using SPSS 25.0, descriptive statistics were computed to determine the composition of the sample with respect to students’ sport background. Chi-square tests were performed to examine associations between students’ sport background and demographic characteristics (sex, age, and year in the medical program). Analysis of variance and correlational analysis were used to examine relationships between students’ sport background, their perceptions of the learning environment in terms of basic psychological needs satisfaction, and their leisure-time exercise in medical school. Due to differences in group sizes, Hedges’ *g* was used in effect size calculations of mean differences, with the value of 0.2 considered a small effect size, 0.5 a medium effect size, and 0.8 a large effect size [24]. Correlation coefficients were interpreted in the same manner.

## Results

Sport background (type and level of involvement) of students in the study is shown in Tab. 1. Results of chi-square tests indicated no significant associations between students’ sport background and demographic characteristics (all *p*’s > 0.05).

**Table 1:** Sport background of the students in the study

Variable	*n* (%)
*Type of sport in which participated the longest*	
– None	18 (9%)
– Individual sport	84 (42%)
– Team sport	98 (49%)
*Highest level of sport involvement*	
– Recreational non-competitive	51 (28%)
– Competed in intramurals or in a recreational league	18 (10%)
– Competed against athletes from my city/town and nearby communities	38 (21%)
– Competed against athletes from around my province/state/territory	24 (13%)
– Competed against athletes from nearby provinces/states/territories	18 (10%)
– Competed at a National Championship	22 (12%)
– Competed against athletes from a country other than my own or as a member of a national team	11 (6%)

Students perceived the learning environment, on average, as supportive of their basic psychological needs for autonomy, competence, and relatedness. As shown in the second column of Tab. 2, the respective average scores were above the midpoint of 14. Students with a team sport background reported the highest satisfaction of the need for relatedness in the medical program (Tab. 2).

**Table 2:** Means (standard deviations) on the measures of the needs for autonomy, competence, and relatedness in the study sample of medical students

Variable	All (*n* = 200)	None (*n* = 18)	Individual sport (*n* = 84)	Team sport (*n* = 98)
Autonomy	17.41 (2.92)	17.00 (3.74)	17.36 (2.75)	17.52 (2.92)
Competence	18.44 (2.49)	18.44 (2.62)	18.33 (2.63)	18.55 (2.36)
Relatedness	18.59 (3.49)	17.44 (4.06)*	18.12 (3.62)	19.19 (3.16)*

*Indicates a statistically significant mean difference (*p* < 0.05); Hedges’ *g* = 0.52.

With respect to the highest level of sport involvement (i.e. from recreational to competitive), a significant, although small, positive correlation was observed with the need for autonomy (*r* = 0.15; *p* = 0.041), indicating that with increasing levels of sport involvement students perceived the medical program as more autonomy-supportive. The correlations between the level of sport involvement and the needs for competence and relatedness were positive but non-significant (*r* = 0.06, *p* = 0.421; and *r* = 0.11, *p* = 0.118, respectively).

Only 3.5% (*n* = 7) of students reported not engaging in exercise in medical school: 11.5% (*n* = 23) engaged only in mild exercise; 15.5% (*n* = 31) engaged in mild and moderate exercise; and 52% (*n* = 104) engaged in mild, moderate, and strenuous exercise during a typical week. The correlation between students’ leisure-time exercise scores and the year in the medical program was negative and significant (*r* = −0.17; *p* = 0.017), indicating a decrease in students’ participation in leisure-time exercise with each year in the program.

With respect to sport background, students who had no involvement in sports in their formative years reported engaging in leisure-time exercise in medical school to a significantly lesser extent (*M* = 10.39; *SD* = 8.49) than students who had pursued either team sports (*M* = 16.95; *SD* = 8.99) or individual sports (*M* = 16.54; *SD* = 8.90) (*p* = 0.016). The effect sizes in leisure-time exercise scores of students with no sport background and students with a team or individual sport background were moderately large (Hedges’ *g* = 0.74 and 0.70, respectively). Finally, there was a significant positive correlation between the level of sport involvement prior to medical school and leisure-time exercise in medical school (*r* = 0.303; *p* < 0.001).

## Discussion

The findings indicate that sport background is associated with students’ perceptions of the learning environment and their leisure-time exercise in medical school. First, medical students with a team sport background reported the highest satisfaction of the need for relatedness in the medical program. Second, students who pursued sport at higher levels of involvement (i.e. competitive sport) perceived the medical program as more autonomy-supportive than students who pursued sport at lower levels of involvement (i.e. recreational, non-competitive sport). Third, students’ involvement in leisure-time exercise in medical school decreased over the years in the program; however, students with a sport background engaged in leisure-time exercise to a greater extent than students with no sport background. Below, we elaborate on each of these findings in context of past literature and implications for practice.

First, compared with students with no sport background, students with a team sport background perceived their need for relatedness to be satisfied to a greater degree in the medical program. This finding suggests that students who had pursued team sports in their formative years may perceive their fellow students as teammates rather than as competitors. The application to medical school is a highly competitive process [25]. However, during medical training students are exposed to team-based learning and work in groups when solving clinical problems [5]. This shift in mindset—from perceiving classmates as competition when applying to medical school to collaborating with them in the program—does not happen automatically, and students with a team sport background may be in a better position to mentally adjust to collaborative aspects of the medical school environment. Further research regarding this shift in mindset is warranted as it may provide knowledge to support such a transition.

Next, students who had pursued sport at higher levels of involvement in their formative years perceived the medical program as more autonomy-supportive than students who had lower levels of sport involvement. This finding suggests that, despite the abundance of rules and regulations in medical school, students with a competitive sport background may be better positioned to recognize learning opportunities in the program and view them as personal challenges that they can overcome with deliberate practice and through stepping out of their comfort zone [12, 26], thereby fulfilling their need for autonomy in the program. Additional qualitative research is needed to further understand the reasons for and further implications of this finding.

Neither the type nor the level of sport involvement was linked to students’ perceptions of the learning environment as supportive of their need for competence in the medical program. We speculate that irrespective of sport background, students require support, feedback, and coaching in developing medical competencies [11] as well as in transferring non-academic skills to academic settings (e.g., training in how to manage challenges in their learning) [19].

Finally, with respect to students’ involvement in leisure-time exercise in medical school, we observed a decrease in exercise over the years in the program. An Australian study with medical students and clinicians reported less involvement in exercise among the study participants than before the commencement of their medical training [27]. Taken together, these results are concerning for at least two reasons. First, medical students and physicians who engage in regular exercise are more likely to counsel their patients on healthy lifestyles [28]. Second, exercise has been shown to be effective in coping with stress [29] and helping protect medical students from developing burnout [16–18]. Wellbeing of medical students becomes particularly important as schools are advancing competency-based medical education [30, 31] and its impact on student wellbeing is yet to be determined. Thus, an important question arises as to which curricular initiatives in the medical education reform are likely to be effective in helping students maintain their leisure-time exercise and sport involvement throughout medical school.

The results of this study must be considered in light of the following limitations as well as its strengths. This study used self-reported survey data, which may pose concerns around social desirability bias. However, given the focus of the study on students’ perceptions, the use of self-reported data appears to be feasible. In addition, considering the busy academic schedules of medical students, the survey used in this study had to be kept short. In light of the findings that sport background is associated with students’ perceptions of the learning environment and their leisure-time exercise in medical school, future studies examining other sport-related characteristics (e.g., length of sport involvement and at what age) are warranted. Next, the data in this study come from students from a single medical school. Although our medical school is representative of other medical schools in Canada, generalizability of the findings may be limited. On the other hand, having students exposed to the same learning environment of one medical program allows for examination of the relationships of interest. Finally, it is not our intent to promote the use of sport background status as a reason for admission, but rather to inform medical schools on how students with a sport background may be perceiving the learning environment and the healthy habits these students engage in the program that could be useful to the student population at large.
